# Improving Emergency Department Efficiency by Patient Scheduling Using Deep Reinforcement Learning

**DOI:** 10.3390/healthcare8020077

**Published:** 2020-03-27

**Authors:** Seunghoon Lee, Young Hoon Lee

**Affiliations:** Department of Industrial Engineering, Yonsei University, 50 Yonsei-ro, Seoul 03722, Korea; shbrandonlee@yonsei.ac.kr

**Keywords:** Healthcare management, healthcare operations, patient scheduling, emergency department, reinforcement learning, deep learning

## Abstract

Emergency departments (ED) in hospitals usually suffer from crowdedness and long waiting times for treatment. The complexity of the patient’s path flows and their controls come from the patient’s diverse acute level, personalized treatment process, and interconnected medical staff and resources. One of the factors, which has been controlled, is the dynamic situation change such as the patient’s composition and resources’ availability. The patient’s scheduling is thus complicated in consideration of various factors to achieve ED efficiency. To address this issue, a deep reinforcement learning (RL) is designed and applied in an ED patients’ scheduling process. Before applying the deep RL, the mathematical model and the Markov decision process (MDP) for the ED is presented and formulated. Then, the algorithm of the RL based on deep Q-networks (DQN) is designed to determine the optimal policy for scheduling patients. To evaluate the performance of the deep RL, it is compared with the dispatching rules presented in the study. The deep RL is shown to outperform the dispatching rules in terms of minimizing the weighted waiting time of the patients and the penalty of emergent patients in the suggested scenarios. This study demonstrates the successful implementation of the deep RL for ED applications, particularly in assisting decision-makers under the dynamic environment of an ED.

## 1. Introduction

An emergency department (ED) is a complicated system due to many factors, such as the limitation of medical resources and the patient’s clinical condition, which are interrelated. Additionally, situations occurring in an ED are difficult to anticipate owing to unscheduled patient visits. Thus, sufficient medical resources cannot be prepared in advance. Because of such complexity and unforeseeable situations, errors by decision-makers are highly possible, which may adversely affect the patient’s treatment sequence. Moreover, one wrong decision could increase the waiting time of patients, leading to crowdedness in the ED. Accordingly, if the waiting time of patients in the ED is increased, their medical conditions may deteriorate, which may lead to significant adverse effects.

Patients visiting the ED can be categorized by using a triage process [1,2,3]. According to the Australian triage scale (ATS) and Canadian triage and acuity scale (CTAS), the acuity is divided into levels 1–5 in accordance with the response time needed to alleviate suffering and address life-threating illnesses. Specifically, acuity level 1 is assigned to a patient needing immediate treatment. For acuity levels 2–5, the response time for treating the patient and the severity of the disease are increasingly less critical. Thus, the waiting time for different patients can be crucial depending on the level of severity of the patient’s clinical condition. However, treating patients with high acuity levels first is not always the best option for hospitals, because patients with low acuity levels should not be ignored. Although the increased waiting time may not worsen their clinical conditions, the delay in treatment can cause dissatisfaction in patients with low acuity levels, which can negatively affect the reputation of the hospital. Therefore, the appropriate management of waiting time in the ED requires consideration of all acuity levels.

For several decades, substantial efforts have been made to resolve the crowding caused by the complexities in EDs. For example, governments have introduced performance- and quality-related targets/indicators [4]. Additionally, some hospitals have enacted a fast-track system in which patients are classified as urgent and non-urgent cases and are treated separately [5]. However, problems associated with the waiting time still remain in EDs. 

Many studies have discussed the efficiency of EDs in a variety of ways. In some studies [6,7,8,9,10], the ED was analyzed using a simulation tool, in which the usability and flexibility effectively expressed the complexity in the ED. After analyzing the current ED situations, some alternatives, such as changing the ED layout [9] and the treatment process [10], were presented to improve its efficiency. Additional studies have suggested a mathematical model solved by mixed-integer programming. Such approaches have been used for nurse scheduling [11], physician shift scheduling [12], and operating room scheduling [13]. However, most challenges for the ED environment can be traced to visiting unscheduled patients [14]. 

In this study, deep reinforcement learning (RL) is employed to schedule patients who visit an ED with limited medical resources under a dynamic environment. The deep RL is originated from the combination of the RL and the deep learning. In the deep RL algorithm, the agent takes an action from a candidate of actions at the current state and takes a reward from the environment. When taking the action, the action is selected by a deep neural network. By iterative learning, the agent finds an optimal policy. Despite the fact that the ED is complicated to design due to lots of interconnected factors, by adapting the algorithm to the ED, the decision is able to be made by the optimal policy. 

The rest of this paper is organized as follows. Section 2 presents a literature review and Section 3 describes the ED environment, the corresponding Markov decision process (MDP) framework, and the algorithm of the deep RL designed for the ED as well. Section 4 presents the deep RL results, and its performance is evaluated by a comparison with the dispatching rules. The discussion and conclusion are revealed in Section 5 and Section 6, respectively.

## 2. Literature Review

### 2.1. Improving Emergency Department Efficiency

The use of EDs has significantly increased worldwide. Because the majority of the patients visiting EDs are urgent cases, numerous studies have been conducted to improve their efficiency. For example, Rismanchian and Lee [15] demonstrated that redesigning the layout of an ED can improve its efficiency by decreasing unnecessary movement. In their study, process mining techniques were used to analyze the process of an ED, and a single layout, meeting several objectives, was presented by goal programming. Khadem et al. [16] used a simulation model to suggest a revised ED layout with improvements over current EDs. Lukkarinen [17] approached the efficiency of an ED regarding its capacity, as well as its medical resource efficiency and availability, by suggesting a new layout and changes in the application of medical resources. Wang et al. [9] adopted value stream mapping to design an ED layout with resources assigned by simulation and optimization. 

Another method for improving the efficiency of an ED is to optimize the processing of patients. Yang et al. [18] suggested three alternative triage processes to minimize the ED crowding, and the alternatives were evaluated by three performance measures: patient length of stay (LOS), variability in the patient length of stay (VLOS), and mean time between the arrival time and the starting time for the first consultation (TFC). Shim and Kumar [19] presented a revised process for patients in the ED by adding another payment station and a new short-stay ward, which minimized the LOS. Spaite et al. [20] evaluated and redesigned the patient flow by using a process-improvement team method that reduced the waiting room time, throughput time, and urgent care waiting room time, thereby increasing the patient satisfaction. Khanna et al. [21] identified critical waypoints in the patient flow and estimated the effects of decreasing the delays in the flow in compliance with the national standard. To improve the patient flow and reduce the patient journey time, Jarvis [22] suggested the use of doctor triage, rapid assessment, streaming technology, and the co-location of a primary care clinician. Oh et al. [10] adopted simulation in their analysis and improved the LOS by altering some of the processes in an ED. Additionally, some authors have discussed the efficiency of the ED with lean thinking [23,24]. Sánchez et al. [25] applied lean thinking to improve the patient flow, which eliminated unnecessary actions by patients and staff to improve the experience for all involved. The key to lean thinking is that improvements are made by eliminating waste [26]. Thus, a variety of methodologies and concepts have been employed to improve the efficiency of EDs.

### 2.2. Patient Scheduling

ED improvements related to changes in the layout or addition of medical resources require monetary investments and may not be feasible in all cases. When considering limitations which are space, budget, and other factors, patient scheduling may be the best option for improving the efficiency of an ED in terms of decision-making. The scheduling in an ED can be classified as patient scheduling or medical resource scheduling. Between these types, patient scheduling, specifically the waiting time of patients, can be more easily changed to improve the efficiency of an ED [27]. Diefenbach and Kozan [28] analyzed an ED by using a simulation model with various criteria objectives and subsequently used an optimization model to optimize the objectives. Kırış at el. [29] presented a knowledge-based reactive scheduling system that considered a patient priority, arrival time, flow time, and doctor load to decide the priority of the patients and reduce their waiting times. Daknou at el. [30] proposed a multi-agent-based approach for scheduling patients by using dynamic scheduling, which was solved under diverse pathways and stochastic processing times of the treatments. Some authors have discussed patient scheduling from the perspective of the manufacturing process. Azadehf [31] analyzed patient scheduling in the ED laboratories by using triage factors. In his study, patient scheduling was modeled in the view of flexible open shop scheduling and mixed-integer linear programming. The scheduling was solved by using a genetic algorithm (GA) that was optimized by applying a response surface methodology. Luscombe and Kozan [14] devised two categories of patient bed assignment and task resource allocation, respectively, to minimize the total care time of patients based on the priority dispatching rule, a disjunctive graph method, and a meta-heuristic method. However, few studies have considered patient scheduling in accordance with environmental changes based on RL.

## 3. Emergency Department Model

This section describes the environment of an ED and explains some assumptions used to model the ED. Generally, the ED addresses emergent patients, which is unexpected and unforeseen. The patients visiting the ED are categorized by three arrival modes: walk-in patients, those arriving by ambulance, and those transported by public services [31]. The type of patient is classified as either adult or pediatric, and they are separated to get treatments in different areas. Their acuity is categorized by the triage method, and their clinical conditions are evaluated according to a symptom-oriented classification tool [32]. After triage, the patient is assigned a bed with priority given to cases of high acuity level; thus, some patients may not be assigned beds. The treatment process proceeds depending on the patient’s clinical condition, in which the pathway of the patients is numerous.

To generalize the ED, there are some assumptions as follows. In this model, the arrival mode of the patient is not considered separately. Once the patient arrives at the ED, the arrival mode is disregarded because the patient is not categorized by that mode. As previously mentioned, patients are assigned beds for treatment, and those with low acuity levels are treated without being assigned a bed. However, if the ED is not busy, a bed may be allocated to a patient with a low acuity level. Thus, for generalization, the bed is excluded in the medical resources. The processing time and the acuity levels are independent; thus, the acuity level does not affect the processing time. Additional assumptions are listed below:

- The moving time of the medical resources or patients is not included in the ED.
- The medical ability of the resource is not graded; all are considered to be equal.
- Medical resources can process only one patient at a time.
- Once the acuity level is assigned to the patient, the level does not change during the stay.
- The treatment process pattern and the acuity level of the patient are known after the patient arrives.
- If an available medical resource and the treatment of a patient are matched, the patient is assigned to the resource immediately. Otherwise, the patient must wait.
- A medical resource group can perform several kinds of treatment.

### 3.1. Mathematical Modeling

In this section, the mathematical model for the ED is presented to figure out the key decision-making point in ED to schedule patients. The details of the mathematical model are described in Appendix A. As seen in the model, the main decision-making is selecting a patient for assigning to a medical resource that is idle, $x_{ijk}$. However, from this decision-making viewpoint, this mathematical model approach is limited in terms of direct use for responding to the dynamic environment, as the environment of ED is changed into real-time. Moreover, due to the complexity of the ED, it needs a solution that keeps the complicated modeling at a distance. To address the issues, the RL algorithm can be used. In the next section, the ED model is described as the MDP to apply the RL. 

### 3.2. Markov Decision Process Framework

As mentioned above, in this section, the MDP framework is presented for the ED. An MDP provides a mathematical framework for modeling the decision-making process, which is widely used for the deep RL. 

The MDP is able to be described as state, action, reward, and transition probability, and the key point is to find a policy that an action chosen from a candidate of actions when in the state. At each time t, the state $s_{t}$ is observed and an action a is selected from a set of action at time t, $a_{t}$. After that, $s_{t}$ is changed as $s_{t+1}$ according to the transition probability, a reward at time t, $r_{t}$, is received. However, as the transition probability is not necessarily required for the RL [33,34], state, action, and reward are discussed.

#### 3.2.1. State Formulation

From the standpoint of the ED operation management, the state is defined as the information of patients who wait for the medical resource type to receive treatments. To describe the state at time t, let ${℘}_{gt}$ be a set of patients who waits to be processed on medical resource type g at time t, $A{℘}_{gt}^{au}$ be the sum of patients who have the acuity level au={1,…,AU}, in ${℘}_{gt}$. As motioned above, the medical resource type g can do several types of treatment, ts={1,…,TS}. Then, let $RT_{gt}^{ts}$ be the sum of patients who have the treatment type ts to be processed on the medical resource type g, in ${℘}_{gt}$. Therefore, the state of each resource group g at time t, $S_{t}^{g}$, can be expressed as below.

(1) $$S_{t}^{g}=\left\{\left(\frac{A{℘}_{gt}^{1}}{\left|{℘}_{gt}\right|},\ldots ,\;\frac{A{℘}_{gt}^{AU}}{\left|{℘}_{gt}\right|}\right),\;\left(\frac{RT_{gt}^{1}}{\left|{℘}_{gt}\right|},\ldots ,\;\frac{RT_{gt}^{TS}}{\left|{℘}_{gt}\right|}\right)\;\right\}$$

In Equation (1), each state vector is reflected as the ratio to standardize vector numbers, which is that the vector numbers are expressed between 0 and 1. The reason that the ratio is reflected is that the scale of each element can be different, which may impact the deep learning negatively. Finally, $s_{t}$ is shown in Equation (2).

(2) $$s_{t}=\left\{S_{t}^{1},\ldots S_{t}^{G}\right\}$$

#### 3.2.2. Action Formulation

The aim of this model is to schedule patients in the ED, and hence the decision-making is to select a patient from a waiting list. As per each medical resource group g, there exist ${℘}_{gt}$ and $a_{t}$ is described in Equation (3).

(3) $$a_{t}=\left\{{℘}_{1t},\ldots {℘}_{Gt}\right\}$$

#### 3.2.3. Reward Formulation

In terms of the objective of the model, the model minimizes the weighted waiting time of patients. Therefore, the reward at time t, $r_{t},$ is defined as the sum of the weighted waiting time until the treatment j–1 of the selected patient i as the action, which shows in Equation (4).

(4) $$r_{t}=-1\times c_{i}\left(\sum_{j=1}^{j-1}wt_{ij}\right)$$

The value of the weighted waiting time of the patient as the reward is counted in negative numbers to correspond to the minimization of the objective.

### 3.3. Deep Reinforcement Learning For Emergency Department

#### 3.3.1. Deep Q-Network

In this section, deep Q-network (DQN) is presented on the basis of the MDP framework described above for finding the optimal policy to schedule patients when patients arrive in real-time. 

As the key decision in this ED model is to select a patient and the DQN is suitable for learning certain actions, the DQN is adapted as the RL algorithm. In addition, it shows a more stable performance and better sample efficiency than other methods. 

Watkins [35] introduced the widely used Q-learning, which is the learning of the optimal policy using a Q-function. The Q-function, Q(s,a), predicts the expected value of the future reward with the pairing of the state and action. Mnih et al. [36] developed the DQN combining the Q-function and an artificial neural network (ANN). For approximating the optimal Q-function, the DQN is essentially the same as Q-learning. The difference is that Q-learning uses a deep neural network to solve the problem of difficulty in learning when the environment is complicated. When using a nonlinear function approximator, such as the ANN, to represent the Q-function, the RL cannot converge owing to the correlations between the Q-value and target value that exist in the order of observations. In this case, the policy changes are affected by the small update. To solve this problem, the DQN uses the replay memory M to eliminate the correlations in the order of observations and to rectify the changes. Additionally, an iterative update is used for redressing the Q-value toward the target value, $\underset{a^{\prime }}{\max}Q\left(s^{\prime },a^{\prime }\right)$, where γ is a discount factor that reduces the correlations with the target, that is, the weight θ of the Q-network is updated at iterations, as defined in Equation (5):

(5) $$L\left(\theta \right)=E_{\left(s_{t},a_{t},r_{t},s_{t+1}\right)\sim U\left(M\right)}{\left(y-Q\left(s_{t},a_{t};\theta \right)\right)}^{2},$$

where $\theta^{-}$ is the weight of the target network $Q^{-}$.

#### 3.3.2. Deep RL Algorithm for ED

Based on the aforementioned introduction, the DQN algorithm to schedule patients in the ED is described in Algorithm 1. Let ${\bar{K}}_{gt}$ be a set of idle medical resources group g at time t. At time t, when ${\bar{K}}_{gt}$ and ${℘}_{gt}$ exist simultaneously, patient scheduling is implemented based on the DQN algorithm and the scheduling is executed until one of the sets is empty.
**Algorithm 1.** Scheduling patients in ED based on DQN with experience replay Input: Scheduling problem Output: Parameters θ of a Q-Network1: Initialize replay memory M
2: Initialize Q-Network with weights θ
3: Initialize target network $Q^{-}$ with weights $\;\theta^{-}$
4: for episode = 1, E do5:  for t = 1, T do6:   Patient arrival i with Poisson distribution7:   Get ${\bar{K}}_{gt}$ and ${℘}_{gt}$
8:   if ${\bar{K}}_{gt}\neq \emptyset$ and ${℘}_{gt}\neq \emptyset$ then9:    repeat10:      Observe $s_{t}$
11:      Select a random action $a_{t}\;\left(i\in {℘}_{gt}\right)$ with probability ε greedy policy. Otherwise, $\;\;\;\;\;\;\;\;a_{t}=\underset{a}{\max}Q\left(s_{t},a;\theta \right)$
12:      Execute $a_{t}$ and Observe $r_{t}$, $s_{t+1}$
13:     Save transition $\left(s_{t},a_{t},r_{t},s_{t+1}\right)$ in M
14:      ${\bar{K}}_{gt}\leftarrow {\bar{K}}_{gt}\backslash \left\{k\right\}$
15:      ${℘}_{gt}\leftarrow {℘}_{gt}\backslash \left\{i\right\}\;$
16:    until ${\bar{K}}_{gt}=\emptyset$ or ${℘}_{gt}=\emptyset$
17:   end if18:   if $\left|M\right|\geq \frac{L}{2}$ and t%60=0 then19:      Sampling random minibatch $\left(s_{h},a_{h},r_{h},s_{h+1}\right)$ from M20:      $y_{h}=r_{h}+\gamma \;\underset{a^{\prime }}{\max}Q\left(s_{h+1},a^{\prime };\theta^{-}\right)$
21:     Perform a gradient decent regarding weights θ
22:   end if 23:  end for24:  Update $\theta^{-}=\theta$
25: end for

When scheduling, the agent observes $s_{t}$, which gains the information of current medical resource $S_{t}^{g}$, and executes an action, i.e., selects a patient from ${℘}_{gt}$, considering the information of each patient. To select a patient as the action, ε-greedy policy is adopted, which the patient is selected from ${℘}_{gt}$ randomly. Otherwise, the action is a patient who has the maximum Q-value in ${℘}_{gt}$. Algorithm 2 shows the procedure of the selection of a patient as the action.
**Algorithm 2.** Action selection  Input: $S_{t}^{g}$, $P_{gt}$
 Output: A patient with maximum Q-value1: rn ← random ( ):2:  if rn ≤ϵ then3:   action ← random $\left(P_{gt}\right)$
4:  else5:   action ← maxQvalue $\left(S_{t}^{g},P_{gt}\right)$
6:  end if

**Figure 1:**
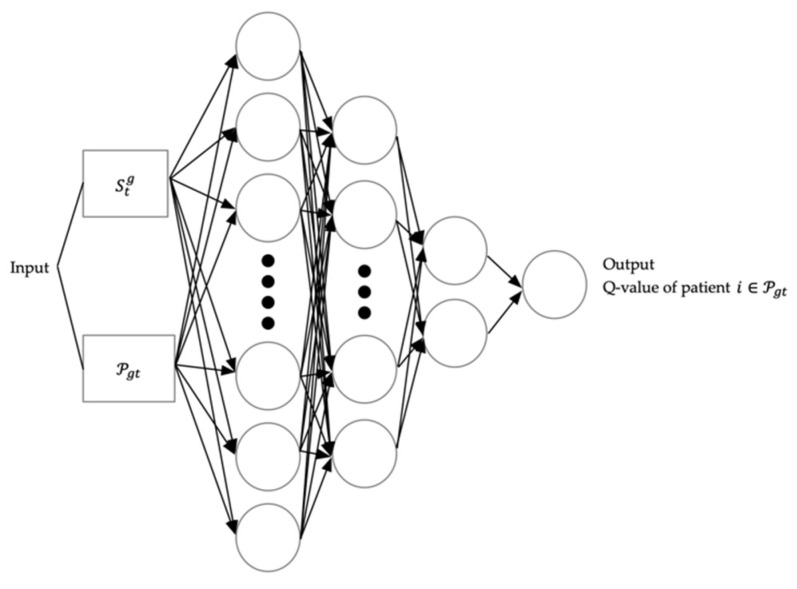
Architecture of the deep *Q*-network to generate Q-value for each patient.

As the way of generating Q-value of an individual patient in ${℘}_{gt}$, $S_{t}^{g}$ and ${℘}_{gt}$ are inserted into ANN as input. As the input, $S_{t}^{g}$ means the information of medical resource  g at time t and ${℘}_{gt}$ stands for the information of each patient in ${℘}_{gt}$. Lots of factors, such as age, sex, arrival time, etc., that a patient has can be considered. In this study, the next factors are chosen for the information of the patient as input: the acuity level, the weighted waiting time, the treatment, $O_{ij}$, and the average processing time of the treatment, $apt_{ij}$. Needless to say, the acuity level and the waiting time of the patient constitute a crucial factor. In addition, the treatment and its average processing time are selected as the factors due to the diverse treatment patterns of patients. The actual processing time of the treatment is unknown unless the patient’s treatment is assigned to the medical resource and is finished, which is why the average processing time is employed. Figure 1 shows the architecture of the deep Q-network.

The next state is observed by the agent and $r_{t}$ is the weighted waiting time of the patient. Transition $\left(s_{t},a_{t},r_{t},s_{t+1}\right)$ is stored and L is the maximum size of the replay memory, in which the old transition is removed once the number of the transition reaches the maximum size, attaining over half the size of the memory. Then, every hour, the loss is calculated before a gradient descent is performed. Lastly, the weight is updated after one episode is finished. 

**Figure 2:**
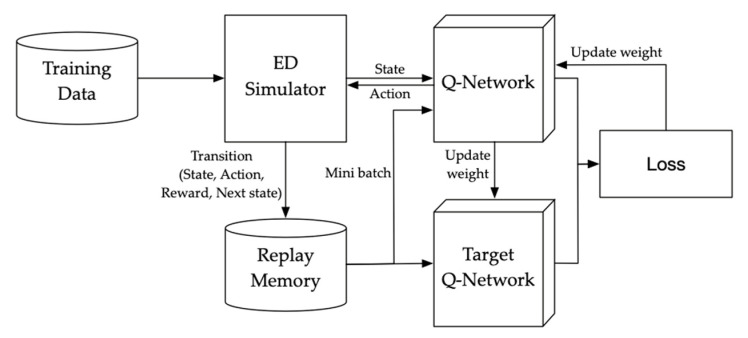
Framework of the deep Q-network for training

In Figure 2, the framework of the deep Q-network for training is shown. A discrete event simulator is employed to design the environment of the ED. Iterations that the simulator deliveries a state and gets an action, and that the Q-network is learned are implemented with the training data. The weight of Q-network is updated at every certain point during the iterations.

## 4. Computational Experiment

### 4.1. Patient Treatment Pattern 

A treatment pattern denotes a patient’s treatment process and patients visiting the ED have different clinical conditions. Depending on the clinical condition, the treatment pattern that the patients receive can be different. This idea is introduced in order to express a series of the treatment process of the patients in the ED.

Rismanchian and Lee [37] analyzed the ED of S hospital in Seoul, South Korea by using a process mining technique with the data of 11,357 patients visiting the ED for 2 months. By using the Disco tool for mining event logs, they identified 11 patterns covering 77% of the treatment processes conducted in the ED, in which the patterns are shown in Table 1. The Basic processes are triage, registration, and evaluation. Depending on the result of the evaluation, the next treatment is varied. The patterns are largely into two groups: discharge and admission. However, in the view of the ED, the admission is regarded as the discharge as well. In Table 2, the medical resources and the time distribution for each treatment are shown. Time distribution from [37] is cited. However, medical resources existing in the ED were not considered in [37]. Therefore, based on a series of unstructured interviews with an intern who worked in the ED, the medical resources were assumed, and the time distribution was revised accordingly. 

Four types of medical resources are considered and devoted to the assigned treatment: doctor, nurse, X-ray technician, and computed tomography (CT) scan technician. Among them, the doctor works in triage and evaluation, and the nurse is in charge of registration, laboratory, consultation, discharge, and admission. In the laboratory case, it is assumed that the laboratory’s capacity is unlimited, which is affected by the number of nurses.

### 4.2. Result

This section presents the results of the RL designed for the ED and a comparison of the conventional and new dispatching rules. The RL was coded in Python with Keras API and was run on a 3.2 GHz Intel i7 with 16 GB RAM. For designing the deep Q-network, a fully connected ANN architecture is employed. The total hidden layers are 3 and the number of the nodes for each layer is 64, 32, 2, respectively. As an activation function, the leaky rectified linear unit is used for all layers. To test the RL for the ED, the experiment was designed as follows. Before comparing the dispatching rules, the RL needs to be trained by the train data set and the hyperparameters used in the deep RL for the experiment are presented in Table 3. Setting the value of the hyperparameters is critical, as they decide the performance of the deep Q-network. However, finding the optimal value is challenging because of the large search space of the hyperparameters. The values in Table 3 were found by conducting a random search [38], which yielded the best performance. 

The patient’s arrival was occurred in accordance with Poisson’s process, λ = 7 which derived from 11,357 historical records of patients visiting the ED within two months. The actual processing time of each treatment was generated by the time distribution at iterations. As mentioned previously, the value of the waiting time differs according to the acuity level; therefore, the waiting time was counted with the weight represented in Table 4. For example, when a patient with an acuity 1 level waits for 1 min, the waiting time is counted as 30 min. In Table 4, the weighted acuity represents the priority of the acuity level, and the ratio is meant to be the frequency of the occurrence of the acuity level in the experiment. After the learning, to evaluate the performance of the RL, the typical and new dispatching rules designed for the ED were suggested in Table 5. 

At the decision-making moment, selecting a patient from a waiting list is made by the rules. To be specific, FCFS selects a patient who arrived at the earliest from a waiting list. The rule of SRPT chooses a patient who has the minimum of $\overset{n_{i}}{\underset{j}{\sum }}apt_{ij}$ from a waiting list. CR is calculated, $\overset{n_{i}}{\underset{j=1}{\sum }}apt_{ij}-t/\overset{n_{i}}{\underset{j}{\sum }}apt_{ij}$, and a patient who has the minimum CR value is selected. If the value calculated by CR is negative, then the patient’s treatment is delayed. AS is calculated in consideration of the weighted acuity and SRPT of patient i: $\left(wk_{1}+wk_{2}\times wa_{i}\right)\times \overset{n_{i}}{\underset{j}{\sum }}apt_{ij}$, where $wk_{1}$ and $wk_{2}$ are the weights. In the same manner, AW is calculated as $\left(wk_{1}+wk_{2}\times wa_{i}\right)\times c_{i}\cdot \left(t-ar_{i}-\overset{j-1}{\underset{j=1}{\sum }}pt_{ij}\right)$. Lastly, the AA first selects a patient with a high acuity level and then considers the earliest arrival time as a secondary factor if patients having the same acuity level are waiting for treatment. In the experiment, $wk_{1}$ and $wk_{2}$ were set as 6 and 4, respectively. It showed good performance in the experiments for demonstrating the validity of the rules.

To evaluate the deep RL performance, the experiment considered four scenarios with unique patient arrival rates. In scenarios 1–4, the patient arrives at the ED according to the Poisson process at λ = 7, 8, 9, and 10 per hour, respectively. Although the normal arrival rate is 7, the RL is tested to evaluate the performance in response to harsh conditions. The medical resources are classified at different levels daily in accordance with the expected workload. For example, the numbers of medical resources are high for some crowded time windows. However, for the analysis of this study, the number of medical resources is constant for all scenarios, i.e., {3, 5, 1, 1}, from the doctor to the CT technician. 

**Figure 3:**
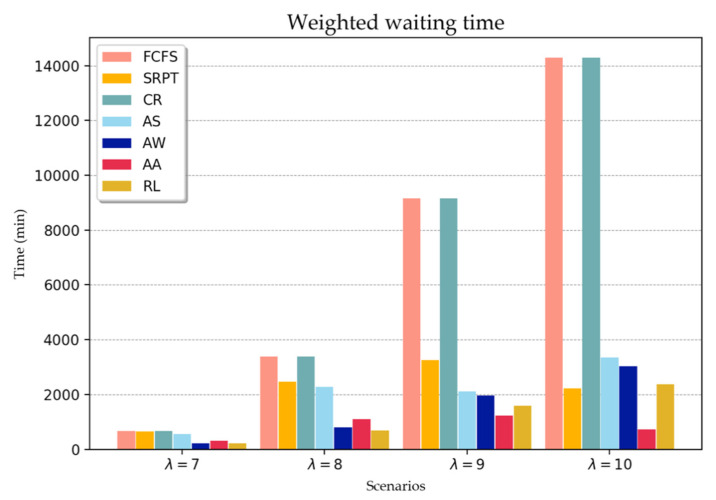
Comparison between conventional dispatching rules and those using RL.

For each scenario, 50 independent instances are generated, with each instance equivalent to 14 days of patient arrival in the ED. When running the simulation, a one-day warm-up period was set to avoid the initial bias that could have affected the performance. Figure 3 illustrates the comparison of the RL and the dispatching rules against the weighted waiting time of the patient. All values were calculated based on the average results of 50 separate instances in each scenario. In a normal situation, scenario 1, the performance of the RL and dispatching rule are not largely different. In particular, the AW rule demonstrates almost the same performance as the RL. In the rest of the scenarios, the acuity-based rules (AS, AW, AA, and RL) outperformed the other rules, except for scenario 4. It is interesting to note that in scenarios 3 and 4, the result of the AA rules yielded better objective values than the RL. However, because the rule selects the patient primarily based on the acuity level, and secondarily based on the arrival time, patients with an acuity level of 5 are rarely discharged. In terms of the average waiting time, the wait for patients with acuity levels 1 and 2 was approximately seven times more than that using RL. In scenario 4, the objective value of the SRPT was lower than that of the RL, which was 146 min. Although the SRPT was better than RL in terms of the objective perspective, the average waiting time of patients with acuity levels 1 and 2 using RL was considerably shorter than that using SRPT. The waiting time and the number of discharged patients according to the acuity level are presented in Table A1. 

To further evaluate the dispatching rules and the RL, an additional performance index is introduced. As previously mentioned, patients who have high acuity levels are required immediate treatment, that is, the shorter waiting time for patients can result in alleviated suffering and avoid deterioration of their medical conditions. To evaluate this, the thresholds for patients assigned acuity levels 1 and 2 are defined as 60 min 180 min, respectively, plus the total average processing time of the treatment. On the basis of this threshold, the penalty patient was calculated in the following manner: The number of patients assigned acuity levels 1 and 2 who remain over the threshold is divided by the number of discharged patients assigned acuity levels 1 and 2. Table 6 presents the experimental results in terms of penalty patients and RL for patients promptly assigned acuity levels 1 and 2 for all scenarios. Less than 2% of the patients violated the threshold when the patients were selected by using RL. The AW rule exhibited almost the same performance as RL in scenario 1. However, when the patient arrival rate increased, the number of penalty patients based on AW was larger than that using RL. In scenario 4, the selection of patients based on FCFS and the CR did not guarantee patients with acuity levels 1 and 2 to be under the threshold. A comparison of AS and AW revealed that the rule of the former produced more penalty patients than that of the latter for the first two scenarios. However, the number of penalty patients based on AW was less than that in the last two scenarios.

## 5. Discussion

In a crowded ED and under unexpected situations, decision-makers may encounter difficulty and challenges in the decision-making process; thus, providing them with a rule or framework to facilitate this task is highly valuable. Decision-making is more critical in the ED than in any other field because the decision can affect the life of a patient. Particularly in crowded conditions, decision-makers may make undesirable decisions because they cannot anticipate future situations. However, the effects of such decision-making processes can be long-term, which can result in adverse outcomes. The result presented in this study indicates that the RL method assists decision-makers in making proper decisions in crowed situations. Overall, the performance of RL was better than that of the currently used dispatch rules. With the presented RL, patients with high acuity levels rarely violated the threshold. The purpose of the AA rule is to discharge as many patients as possible with high acuity levels to enable fewer patients with low acuity levels to be discharged. The results of scenarios 3 and 4 demonstrated that one patient with acuity level 5 was discharged or could not be discharged at all. Abandoning the opportunity for discharging the patients with low acuity levels, the number of penalty patients of the AA was larger than the RL; the waiting time of patients with high acuity levels was also longer than that using RL. On the contrary, selecting the patients based on RL showed that the proper number of patients at all acuity levels were discharged. Additionally, the number of discharged patients at acuity levels 1 and 2 was the largest among the rules. Similar trends were observed in other scenarios, implying that RL can assist decision-makers in the proper selection of patients in normal and extreme situations.

The patient scheduling field has been studied mainly under static environments rather than dynamic environments. Moreover, although the environments were dynamic, it has been difficult to fully cover dynamic situations. An electronic medical record (EMR) system has been introduced to the ED of hospitals, in which patients’ data are collected in a database system. Thus, the data represents the ED’s own characteristics and the discussed algorithm is able to learn a policy to schedule patients in the ED on a basis of them. According to the characteristics, the hyperparameters of the algorithm need to be tuned for a better solution. On top of that, it is necessary that the algorithm with the learned policy is required to run the simulation with the random data to check the validation. Once the algorithm is employed, it is essential that the performance is monitored persistently by clinicians and engineers at the same time. To embrace a variety of changes in the situation of the ED, the algorithm needs to continuously learned and is updated on a certain point basis. Approaching this perspective, the decision-makers are able to be supported by the algorithm when making decisions to schedule patients under dynamic and crowding environments.

This study is limited by the assumptions made as a result of the lack of data on medical resources. Although the number of medical resources is flexible according to the time window, the information was not available in the literature. Generally, although the number of medical resources is limited, they are allocated at the proper level to avoid crowding in the ED. To obtain the estimates for this information, the simulation was run several times. As further limitations, the ED model in the study contained major elements, while other departments connected to the ED, such as operating rooms, were excluded. Furthermore, the interruptions of the treatments were not considered in the model. Moreover, the beds were not modeled to consider patients with low acuity levels who do not receive treatment beds. Lastly, the skill levels of the resources were not considered; the role of a resource for taking charge of treatment differs according to the skill level. Even if the aforementioned assumptions affected the accuracy of the model, the ED components included in this study are sufficient to represent the testing based on RL. 

This study can be expanded to consider ambulance diversion which affects the crowding of the ED. Integrating the EDs and the arrivals by ambulance can more realistically represent the crowding conditions of the ED. Moreover, this study can be addressed in a multi-agent RL perspective incorporating other factors, such as operating rooms. As another extension, with more advanced technologies, if the correlation between factors of the patient such as age, sex, clinical condition, etc., the patient’s life, and the waiting time is able to be analyzed, more practical solutions can be suggested.

## 6. Conclusions

The crowding of EDs has become a significant problem internationally in the last decade. Not only increasing demand of the ED, but also a shortage of beds in the ED and insufficient medical staff can cause such crowding. Crowding in ED is regarded as a critical problem because it leads to the long waiting time of patients and affects the level of the satisfaction of services. Scheduling patients is one of the methods to improve the efficiency of the ED, which is a more practical method than others such as optimizing the layout of the ED or increasing the resources because it does not require large investments of time or funds. However, patient scheduling is complicated to achieve in the context of ED efficiency due to a variety of factors that are interconnected. It is hard for decision-makers to consider the factors simultaneously during scheduling under the dynamic environment. To address this issue, the deep RL is designed and applied for the ED to schedule patients. In order to apply the deep RL, the mathematical model of the ED is presented. This model catches the main decision-making of the ED for scheduling patients. Then, the MDP is designed for the ED, which formulates state, action, and reward accordingly. Finally, the DQN is used for finding the optimal policy when scheduling. 

In the study, the objective is the weighted waiting time of the patients, such that the waiting time value differs between patients with high and low acuity levels. To evaluate the deep RL performance, dispatching rules were presented for a comparison with the deep RL results. Under diverse scenarios, the deep RL generally outperformed the dispatching rules. In particular, the suggested RL exhibited minimized waiting times for patients with high acuity levels in normal and extreme situations. Furthermore, the number of penalty patients was minimal; thus, the algorithm assures the priority of high acuity levels. The key contribution of this study is that the decision-making in the ED is approached from the view of using the deep RL, and this algorithm can support the tasks of decision-makers for improving the efficiency of the ED in a dynamic environment.

## Figures and Tables

**Table 1:** Patient treatment pattern.

Pattern	Description	Treatment	Description
1	A → B → C → H	A	Triage
2	A → B → C → E → H	B	Registration
3	A → B → C → D→ E→ H	C	Evaluation
4	A → B → C → F → H	D	Laboratory
5	A → B → C → D → E → F → H	E	X-ray
6	A → B → C → D→ H	F	Consultation
7	A → B → C → E→ D→ H	G	CT scan
8	A → B → C → G→ H	H	Discharge
9	A → B → C → G→ E→ F→ I	I	Discharge (admission)
10	A → B → C → D→ I		
11	A → B → C → E→ D→ I		

→: Treatment sequence

**Table 2:** Time distribution of treatments.

Treatment	Description	Time Distribution (Min)	Resource
A	Triage	Expo (7)	Doctor
B	Registration	Expo (5.5)	Nurse
C	Evaluation	Normal (μ=14, σ=6)	Doctor
D	Laboratory	Normal (μ=35, σ=15)	Nurse
E	X-ray	Expo (12)	X-ray technician
F	Consultation	Normal (μ=15, σ=8)	Nurse
G	CT scan	Normal (μ=29, σ=14)	CT technician
H	Discharge	30	Nurse
I	Discharge (admission)	Expo (3)	Nurse

**Table 3:** Hyperparameters.

Hyperparameter	Value
Learning rate	$10^{-7}$
Epsilon	1
Epsilon decay	0.999
Epsilon min	$10^{-8}$
Discount factor	0.9999
Size of memory	2000
Iteration	1000
Batch size	100
Optimizer	Adam

**Table 4:** Weighted waiting time, weight acuity, and ratio of occurrence.

Acuity Level	Weighted Waiting Time (min)	Weighted Acuity (wa)	Ratio (%)
1	30	5	10
2	15	4	30
3	1	3	40
4	1	2	10
5	1	1	10

**Table 5:** Dispatching rules.

Dispatching Rule	Description
First Come, First Served (FCFS)	Select the patient arriving at the earliest time in the ED
Shortest Remaining Processing Time (SRPT)	Select a patient having the shortest remaining average process time
Critical Ratio (CR)	Select a patient by dividing the actual time remaining for a particular treatment by the estimated time required for the entire treatment process
Acuity and SRPT (AS)	Select a patient based on the linear combination of two weighted variables: weighted acuity and SRPT
Acuity and Waiting Time (AW)	Select a patient based on the linear combination of two weighted variables: weighted acuity and weighted waiting time
Acuity and Arrival Time (AA)	Select a patient considering the high acuity level and earliest arrival time as primary and secondary factors, respectively

**Table 6:** Number of penalty patients (%).

Scenario	FCFS	SRPT	CR	AS	AW	AA	RL
1	38.59	24.27	38.59	11.08	0.95	7.19	0.90
2	95.49	40.42	95.49	18.18	4.01	19.93	1.36
3	99.91	38.00	99.91	19.93	32.46	21.14	1.56
4	100	31.40	100	30.38	61.79	13.89	1.64

## Appendix A

The notations are described, and the mathematical model for the ED is proposed in consideration of the assumptions explained in Section 3. Consider a set of patients ℘ to be scheduled in the ED. The treatment processes are assigned to patient i according to the clinical condition in a fixed sequence. Each treatment j has a set of the eligible medical resource type g and a treatment j is able to be processed on a medical resource $k\in K_{g}$ with processing time $pt_{ij}$. In this model, it assigns each treatment of patients to one of the alternative medical resources and decides the starting time of the treatments of the patients. Some additional notations and the formulation of the ED are presented as following.

Set:	
$K_{g}$	A set of medical resource type g
℘	A set of patients
Indices:	
i	individual patient
j	individual treatment
k	individual resource
g	medical resource type g=1,…,G
Parameters:	
$ar_{i}$	Arrival time of patient i
$au_{i}$	Acuity level of patient i
$wa_{i}$	Weighted acuity level of patient i
$n_{i}$	Total number of treatments for patient i
$pt_{ij}$	Actual processing time of treatment j for patient i
H	Large number
$O_{ij}$	Treatment j for patient i
$c_{i}\;$	Weight for wait time of patient i
Decision variable:	
$wt_{ij}$	Waiting time for $O_{ij}$ to be started
$st_{ij}\;$	Commencing time for $O_{ij}$
$et_{ij}\;$	Completion time for $O_{ij}$
$x_{ijk}$	1 if treatment j for patient i is assigned to resource k, 0 otherwise

The goal of the model is to minimize the weighted waiting time of the patient’s stay in the ED as shown in Equation (A1), by assigning a patient to the appropriate medical resource. As mentioned earlier, each patient is categorized by the triage method and is assigned an acuity level. The value of the waiting time can be different according to the patient’s acuity level. Therefore, in light of this characteristic, the waiting time of patients with higher acuity levels is weighted larger even when their wait time may be the same as those of patients with low acuity levels.

(A1) $$Objective=\underset{\;}{\min}\frac{\sum_{i\in ℘}c_{i}\left(\sum_{j=1}^{n_{i}}wt_{ij}\right)}{\left|℘\right|}$$

(A2) $$\sum_{k\in K_{g}}x_{ijk}=1,\;\forall \;j=1,\ldots ,n_{i},\;i\in ℘$$

(A3) $$st_{ij}\geq st_{i\left(j-1\right)}+\sum_{k\in K_{g}}pt_{i\left(j-1\right)}\cdot x_{ijk},\;\forall \;j=2,\ldots ,n_{i},\;i\in ℘$$

(A4) $$wt_{ij}=st_{ij}-ar_{i}\;,\;\forall \;i,\;\;j=1$$

(A5) $$wt_{ij}=st_{ij}-et_{i\left(j-1\right)}+wt_{i\left(j-1\right)}\;,\;\forall \;i,\;\;j>1$$

(A6) $$st_{ij}\geq st_{i^{\prime }j^{\prime }}+p_{i^{\prime }j^{\prime }}-\left(1-x_{ijk}\right)\cdot H\;,\;\forall j=1,\ldots ,n_{i},\;j^{\prime }=1,\ldots ,n_{i^{\prime }},\;i,\;i^{\prime }\in ℘\;s.t.\;\;k\in K_{g}$$

(A7) $$x_{ijk}\in \left\{0,\;1\right\},\;\forall j=1,\ldots ,n_{i},\;\;i\in ℘,k\in K_{g}$$

Constraints (A2) mean each treatment is assigned to one of the entitled medical resources. Constraints (A3) guarantee the precedence relations between consecutive treatments of the same patient. Constraints (A4) and (A5) are calculating the waiting time between the patient’s treatments. Constraints (A6) ensures to prevent the overlapping of the treatments on the same resource. Constraints (A7) are the decision variable.

**Table A1:** Waiting time and number of discharged patients (min/person).

Scenario	Rule	Acuity 1	Acuity 2	Acuity 3	Acuity 4	Acuity 5
1 (λ=7)	FCFS	110.94 (203.78)	110.78 (306.54)	111.22 (1327.3)	110.94 (104.08)	110.36 (100.98)
	SRPT	107.6 (203.74)	106.86 (306.72)	107.98 (1327.24)	104.92 (104.16)	108.7 (101.06)
	CR	182.1 (203.78)	181.6 (306.54)	182.2 (1327.3)	181.58 (104.08)	182 (100.98)
	AS	75.52 (204.3)	86.3 (306.7)	143.66 (1325.56)	250.74 (103.46)	390.12 (99.24)
	AW	21.76 (204.94)	26.24 (307.86)	122.64 (1326.96)	147.92 (104.0)	181.68 (100.52)
	AA	40.2 (204.76)	41.3 (307.78)	78.98 (1330.6)	233.06 (103.3)	614.28 (98.2)
	RL	20.86 (204.96)	20.46 (307.9)	129.7 (1326.68)	130.42 (104.02)	126.92 (100.92)
2 (λ=8)	FCFS	567.38 (218.66)	567.42 (334.78)	568.5 (1450.14)	565.62 (111.26)	570.06 (110.44)
	SRPT	422.68 (219.22)	401.9 (336.38)	417.52 (1454.14)	407.68 (111.52)	439.58 (111.26)
	CR	567.38 (218.66)	567.42 (334.78)	568.5 (1450.14)	565.62 (111.26)	570.06 (110.44)
	AS	267.62 (214.64)	353.36 (327.06)	587.1 (1337.84)	1238.38 (87.86)	2804.18 (59.54)
	AW	37.66 (228.76)	60.14 (350.08)	684.8 (1434.86)	845.56 (108.3)	1125.42 (105.02)
	AA	149.18 (226.52)	145.12 (346.76)	225.14 (1496.02)	729.82 (107.48)	6536.8 (38.48)
	RL	25.1 (229.14)	24.86 (351.02)	733.36 (1428.66)	728.24 (109.54)	735.08 (109.02)
3 (λ=9)	FCFS	1521.64 (225.02)	1504.14 (335.76)	1512.7 (1141.18)	1512.08 (113.12)	1507.82 (109.42)
	SRPT	546.48 (228.58)	527.72 (342.8)	544.2 (1473.12)	551.36 (115.76)	561.84 (112.46)
	CR	1521.64 (225.02)	1504.14 (335.76)	1512.7 (1441.18)	1512.08 (113.12)	1507.82 (109.42)
	AS	127.64 (224.1)	208.98 (331.96)	1466.26 (1119.32)	1213.5 (62.34)	4157.5 (17.42)
	AW	65.76 (259.68)	124.58 (386.64)	1870.44 (1385.42)	2338.98 (102.8)	3118.94 (89.94)
	AA	168.58 (245.56)	177.26 (366.76)	273.36 (1538.74)	1479.2 (31.0)	53.96(0.02)
	RL	26.12 (260.6)	26.3 (389.96)	2011.36 (1363.76)	2009.88 (107.12)	2007.16 (103.66)
4 (λ=10)	FCFS	2387.9 (217.52)	2411.12 (329.64)	2400.24 (1436.3)	2401.58 (108.86)	2415.3 (109.34)
	SRPT	379.54 (224.64)	360.18 (338.76)	375.42 (1483.54)	406.06 (112.06)	364.5 (113.22)
	CR	2387.9 (217.52)	2411.12 (329.64)	2400.24 (1436.3)	2401.58 (108.86)	2415.3 (109.34)
	AS	161.46 (241.48)	675.16 (340.8)	505.58 (955.06)	2225.0 (36.36)	5214.66 (7.72)
	AW	90.76 (279.48)	185.22 (420.76)	2987.24 (1336.74)	3736.62 (90.72)	5140.72 (74.64)
	AA	82.26 (243.96)	79.62 (371.26)	297.34 (1433.26)	540.52 (30.28)	0 (0)
	RL	27.02 (281.5)	27.6 (426.8)	3213.78 (1298.32)	3189.56 (97.68)	3228.82 (98.7)
